# Polygenic impact of common genetic risk loci for Alzheimer’s disease on cerebral blood flow in young individuals

**DOI:** 10.1038/s41598-018-36820-3

**Published:** 2019-01-24

**Authors:** Hannah L. Chandler, Richard G. Wise, Kevin Murphy, Katherine E. Tansey, David E. J. Linden, Thomas M. Lancaster

**Affiliations:** 1 Cardiff University Brain Research Imaging Centre (CUBRIC), School of Psychology, Cardiff University, Cardiff, UK; 2 Neuroscience and Mental Health Research Institute, Cardiff University, Cardiff, UK; 3 MRC Centre for Neuropsychiatric Genetics and Genomics, Institute of Psychological Medicine and Clinical Neurosciences, Cardiff School of Medicine, Cardiff University, Cardiff, UK; 4 Cardiff University Brain Research Imaging Centre (CUBRIC), School of Physics and Astronomy, Cardiff University, Cardiff, UK; 5 Dementia Research Institute, School of Medicine, Cardiff University, Cardiff, UK

## Abstract

Genome-wide association studies (GWAS) show that many common alleles confer risk for developing Alzheimer’s disease (AD). These risk loci may contribute to MRI alterations in young individuals, preceding the clinical manifestations of AD. Prior evidence identifies vascular dysregulation as the earliest marker of disease progression. However, it remains unclear whether cerebrovascular function (measured via grey-matter cerebral blood flow (gmCBF)) is altered in young individuals with increased AD genetic risk. We establish relationships between gmCBF with *APOE* and AD polygenic risk score in a young cohort (N = 75; aged: 19–32). Genetic risk was assessed via a) possessing at least one copy of the *APOE* ɛ4 allele and b) a polygenic risk score (AD-PRS) estimated from AD-GWAS. We observed a reduction in gmCBF in *APOE* ɛ4 carriers and a negative relationship between AD-PRS and gmCBF. We further found regional reductions in gmCBF in individuals with higher AD-PRS across the frontal cortex (P_FWE_ < 0.05). Our findings suggest that a larger burden of AD common genetic risk alleles is associated with attenuated cerebrovascular function, during young adulthood. These results suggest that cerebral vasculature is a mechanism by which AD risk alleles confer susceptibility.

## Introduction

Genome-wide association studies (GWAS) have demonstrated that Alzheimer’s disease (AD) has a highly polygenic basis, where potentially thousands of common risk alleles confer susceptibility^1^. Although the polygenic architecture has shown utility in predicting AD^2,3^, the neurobiological mechanisms by which these loci confer risk remain poorly understood. Imaging genetics can reveal the neurobiological mechanisms by which genetic loci confer risk for AD. For example, several studies show that common variation in the *APOE*, *CLU*, *PICALM and BIN1* genes are associated with AD may influence the structure and function of the human brain decades before disease onset^4–10^. Although these AD risk alleles are associated with changes in brain volume and blood oxygen level dependent (BOLD)^11^, little is known about how they are linked to global cerebrovascular function in young asymptomatic individuals.

Cerebrovascular function has been linked to key AD biological pathways such as i) cholesterol metabolism and blood brain barrier permeability^12,13^ and ii) arteriosclerosis of vessels^14^. Pivotally, recent evidence suggests that vascular dysregulation is the earliest marker of AD progression, in a cohort of 40–70 year old individuals^15^. In addition, several initial studies have demonstrated that the presence of AD risk alleles such as *APOE* ε4 or familial risk of AD are associated with alterations in neurovasculature, where older individuals with risk for developing AD show reduced cerebral blood flow (CBF)^16–19^. However, no study has yet explored how the comprehensive polygenetic architecture of AD is linked to cerebrovascular function in young asymptomatic individuals. The observations from such a study could identify links between genetic risk loci that may disrupt cerebrovascular health later in life as a mechanism of increased AD susceptibility.

In the current study, we sought to extend the findings of recent evidence examining vascular dysregulation as one of the earliest markers of disease progression in AD^15^ by examining vascular effects in young individuals who have increased AD genetic risk. Specifically, we sought to explore the cumulative effects of both *APOE* and all the other common genetic risk loci (as measured using an AD-PRS) identified by recent GWAS^1^. We had two key hypotheses. First, given that reductions in grey matter CBF (gmCBF) have been observed in older individuals who possess at least one copy of the ɛ4 allele, we predict this effect would also be observed in young ɛ4 carriers. Second, we anticipated that as cumulative risk for developing AD increased, we would also observe a decrease in gmCBF. Moreover, we suggest that the alterations in gmCBF observed in AD populations^15^ may be partly influenced by common genetic AD risk factors. We anticipate that the combined effect of these risk alleles can be observed in asymptomatic individuals.

## Results

### Demographic analysis

Consistent with prior epidemiological reports^20^, females had higher resting gmCBF than males (Table 1). There were no significant associations between AD-PRS and age (P > 0.09, in all cases) or gender (P > 0.1, in all cases). Grey matter CBF, GMV and AD-PRS were normally distributed (Shapiro test; P > 0.1 in all cases). *APOE* ɛ4 status was not related to GMV, composite cognition or any of the seven cognitive subdomain measures (P > 0.1, in all cases; see Table 2). Similarly, AD-PRS was also not related to GMV or any cognitive measures (P > 0.1, in all cases).

**Table 1:** Statistics for each predictor in the model.

Predictor	Standardized Estimate	Standard Error	t-value	P value
AD-PRS	−0.232	0.751	−2.201	0.031
*APOE* (ɛ4)	−0.213	1.633	−2.055	0.044
Age	−0.085	0.259	−0.737	0.464
Gender (Male)	−0.356	1.708	−3.046	0.003
GMV	0.032	0.899	0.250	0.803

Linear regression model of whole grey matter CBF. Estimate = adjusted beta coefficients. GMV = grey matter volume, AD-PRS = Alzheimer’s disease polygenic risk score.

**Table 2:** Descriptive/demographic statistics for individuals included in final linear regression models (n = 75).

Gender	*APOE* ɛ4 (−) n = 54		*APOE* ɛ4 (+) n = 21		*P*
	M = 19/F = 35		M = 9/F = 12	X^2^ = 0.12	0.726
	Mean	SD	Mean	SD	
Age	23.759	3.192	23.952	3.170	0.814
GMV	0.767	0.011	0.768	0.011	0.719
AD-PRS	−0.075	0.935	0.192	1.154	0.351
SoP	68.764	23.639	76.042	19.612	0.185
AV	44.214	26.689	51.118	22.990	0.276
WM	56.411	21.998	58.161	23.945	0.775
VrblLrng	51.480	28.071	47.137	29.272	0.566
VisLrng	66.795	25.647	62.667	27.922	0.563
RPS	54.906	28.815	62.205	24.900	0.287
SC	45.295	25.777	47.147	30.897	0.810
Comp	59.971	23.479	63.183	18.739	0.543

X^2^ = chi squared test for gender. All other demographics were tested via two-sample t-test. SD = standard deviation. GMV = grey matter volume; AD-PRS = Alzheimer’s disease polygenic risk score (z-scores). SoP = Speed of Processing; AV = Attention Vigilance; WM = Working Memory; VrblLrng = Verbal Learning; VisLrng = Visual Learning; RPS = Reasoning and Problem Solving; SC = Social Cognition; Comp = Composite Cognition Score.

### Genetic analysis of whole brain gmCBF

**Figure 1:**
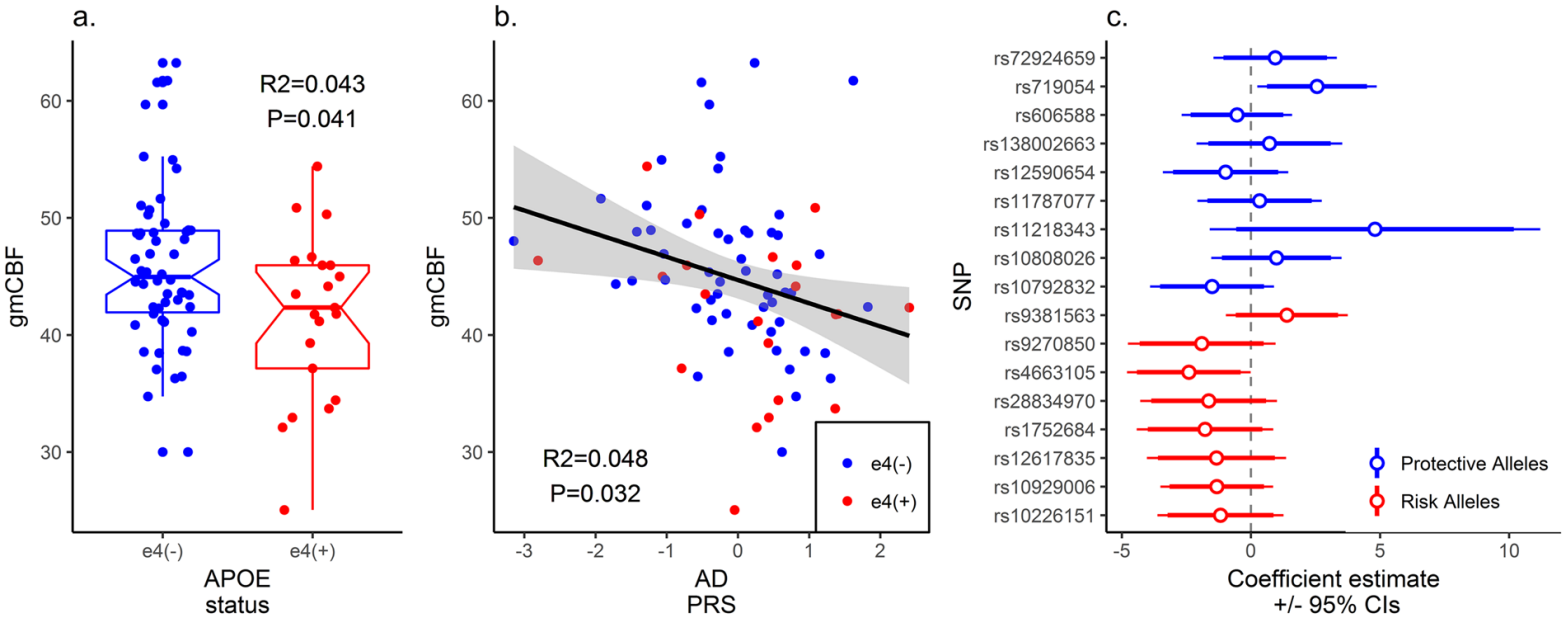
Results for both the *APOE* status group difference in gmCBF and the association between AD-PRS and gmCBF. (**a**) Total gmCBF (grey matter cerebral blood flow) stratified by presence of *APOE* ε4 isoform; (**b**) association between gmCBF and AD-PRS. *R*^2^ and p-values estimated from linear regression, controlling for age, gender, GMV and PRS (in (**a**)) or *APOE* (in (**b**)). (**c**) Reflects the individual contributions to gmCBF for all SNPs within the AD-PRS (N_SNPS_ = 17), controlling for covariates. CIs = confidence intervals.

The linear regression revealed a significant association between gmCBF and both *APOE* (β = −0.213, P = 0.044) and AD-PRS (β = −0.232, P = 0.031) controlling for age, gender and GMV (see Table 1 for full regression model; Fig. 1a,b for effect size estimates). To control for population stratification effects, we repeated the regression, including the first five principle components (PCAs) from the LD-pruned genotype data. Including these additional five covariates did not significantly affect the association between gmCBF and AD-PRS (β = −0.251, P = 0.022). A SNP-wise post-hoc analysis of AD-PRS showed that the majority of alleles over-represented in the AD population (risk alleles) were associated with reduced gmCBF, while alleles over-represented in the healthy population (protective alleles) were mostly associated with increased gmCBF (Fig. 1c). This assumption was confirmed via a sign test (13/17 SNPs with expected direction, P < 0.05). Consistent with an additive polygenic model, no single SNP appeared to drive the AD-PRS effects. Post-hoc analysis of AD-PRS with progressively liberal thresholds demonstrated similar relationships at P_T_ < 5 × 10^−6^ (β = −0.22, P = 0.045) but not at P_T_ < 5 × 10^−4^ (β = −0.03, P = 0.74) and P_T_ < 5 × 10^−2^ (β = 0.01, P = 0.91).

### Genetic analysis of grey matter density

We found no other evidence of association between APOE status or AD-PRS and grey matter density in a voxel-based search across the brain (P_FWE_ > 0.1, in all cases).

### Post-hoc exploratory regional analysis

**Figure 2:**
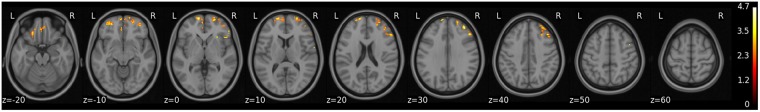
Voxel wide analysis of gmCBF for AD-PRS. Regional association between gmCBF and AD-PRS across all grey matter. All clusters that remain (red-yellow) reflect voxels that survived correction across the whole brain (using threshold free cluster enhancement) with permutation testing (N = 5000) (P_FWE_ < 0.05). Color-bar represents regional t-statistic.

We conducted a post-hoc exploratory voxel-wide analysis across gmCBF. All voxel-based analyses were estimated accounting for both *APOE* and AD-PRS (including aforementioned covariates). *APOE* ε4 status was not regionally associated with CBF in any voxels after correction for multiple comparisons (P_FWE_ > 0.05). However, AD-PRS was significantly associated with gmCBF in several prefrontal regions, which were significant after controlling for multiple comparisons (P_FWE_ < 0.05). All clusters were located in the frontal lobe (including the frontal pole, middle frontal gyrus, inferior frontal gyrus, insular, frontal medial cortex & orbitofrontal cortex; see Fig. 2).

## Discussion

We find negative associations between i) *APOE* ε4 status and ii) AD-PRS with gmCBF, providing evidence that an increasing number common AD genetic risk loci is associated with reduction in resting gmCBF in young asymptomatic individuals. These findings support observations between *APOE* and reductions in resting gmCBF in older individuals^21^. We also provide novel evidence that the GWAS significant common AD risk loci identified via GWAS were negatively associated with gmCBF. Our post-hoc observations between AD-PRS in frontal regions conforms with preclinical models demonstrating that reduced frontal gmCBF is linked to genetic risk^22^, and may aid in the understanding of future cognitive decline^23^. Together, these analyses provide support for a broader hypothesis that AD genetic risk loci may confer susceptibility via alterations in cerebrovascular health, decades before the onset of clinical symptoms. This is also evidenced by several AD-PRS imaging studies showing associations between an AD-PRS and MRI based measures of brain health, including macrostructure, white matter microstructure and blood oxygen level dependency (BOLD) during memory processing in the medial temporal lobe^24–28^.

These findings conform to pre-clinical models of AD where functional impairments such as widespread alterations in cerebral blood flow precede grey matter atrophy and/or cognitive impairments^15^. Our findings can also be assessed in light of other recent evidence showing that reduced CBF is associated with a reduction in cognitive function in older individuals^20^. In the current study, we find reduced gmCBF in young asymptomatic individuals suggesting that vascular alterations are present prior to the onset of symptoms, supporting recent research conducted in an older sample^15^. However, putative dynamic relationships between AD-PRS and brain imaging traits linked to AD across the lifespan remain largely untested. Furthermore, it is critical that future research investigates the precise nature of this alteration and assesses the relationship between gmCBF and oxygen dynamics including cerebral metabolic rate of oxygen (CMRO_2_), oxygen extraction fraction (OEF) and oxygen diffusivity (for methods see^29–32^).

Our findings should be taken with the following considerations. We appreciate that while this is a large imaging sample, it is relatively small for a genetic study and further investigations should aim to a) replicate these observations to confirm these associations in younger individuals and b) expand population level imaging protocols to vascular measures. However, considering the moderate impact of both *APOE* and AD-PRS, we suggest that this study was adequately powered to detect the cumulative effect of these risk factors. Lastly, as the *APOE* status and AD-PRS effects were in the same direction, we suggest that cumulative genetic risk may be more broadly related to lower gmCBF. Lastly, as we did not see a relationship between AD-PRS and gmCBF at more liberal p-thresholds, suggesting the precise genetic architecture of AD that contributes to imaging measures remains unclear.

Our observations highlight possible therapeutic benefits of exercise (and associated increases in CBF) for neurodegenerative disease^33^. While evidence suggests that exercise may improve cerebrovascular health, the longitudinal effects of exercise as a therapeutic tool for AD remain unknown. Furthermore, dietary supplements such as nitrates have been shown to increase CBF in healthy adults^34^. Thus, future research should investigate the beneficial effects of such a supplement for increasing gmCBF in individuals with high genetic risk of developing AD.

In conclusion, we observed an association between reduced CBF and AD genetic risk loci (specifically – evidence for the involvement of *APOE* and independent top GWAS risk loci). Future studies may be able to investigate biologically informed pathways to understand how AD imaging phenotypes are influenced by genetic risk. We suggest that CBF may be a useful tool in understanding how genetic risk for AD may affect the human brain in young individuals and has implications for functional MRI studies that relate BOLD and models of neurovascular coupling.

## Methods

### Participants

One hundred healthy, right-handed individuals were recruited via advertisement from Cardiff University. Participants reported no history of psychiatric or neurological illness, and were not taking any psychotropic medication. The study was approved by the ethics committee of the School of Psychology, Cardiff University. Each participant provided written informed consent. Participants were excluded from the study if they were over 35 (n = 2), failed genotyping quality control (*n* = 12) or failed quality control for the imaging procedure (n = 9). In addition, those who possessed the *APOE* ε2ε4 isoform (n = 2) were also excluded due to the presence of the protective ε2 allele, as previously described^27^. Our final sample consisted of 75 individuals, 54 of whom possessed no copy of the ε4 allele (pooled: ε2ε3 & ε3ε3) and 21 individuals who possessed at least one copy of the ε4 allele (pooled: ε3ε4 & ε4ε4). There was no association between *APOE* status with age, gender, grey matter volume (GMV) or AD-PRS (Table 1). *APOE* status and AD-PRS were entered into the regression model together (see statistical analysis for further details).

### Genotyping and extraction of DNA

Genomic DNA was obtained from saliva using Oragene OG-500 saliva kits (DNA Genotek, Inc., Ontario, Canada). Genotyping was performed using custom Illumina. HumanCoreExome-24 BeadChip genotyping arrays, which contain approximately 500,000 common genetic variants (Illumina, Inc., San Diego, CA). Quality control and imputation were implemented in plink 1.9^35^. Briefly, individuals were excluded for any of the following reasons: 1) ambiguous sex (where samples with undetermined X chromosome heterozygosity were excluded); 2) cryptic relatedness up to third-degree relatives as ascertained using identity by descent; 3) genotyping completeness less than 98%; 4) non-European ethnicity admixture which was determined via population stratification, where samples that clustered outside the CEU HapMap3 population using principal component analysis were excluded); and 5) outliers from an autosomal heterozygosity filter. Single nucleotide polymorphisms (SNPs) were excluded where the minor allele frequency was less than 1%, if the call rate was less than 98%, or if the χ2 test for Hardy-Weinberg equilibrium had a p value less than 1e-6. A total of 233054 genotyped SNPs remained after quality control. Autosomal chromosomes were imputed using the reference panel HRCv1.1 (hrc.r1.1.2016) using a mixed population panel^36^. Phasing was completed using Eagle v2.3^37^ and imputation was performed using Mimimac3^38^. Imputed data was converted to best-guess genotypes^35^ with multi-allelic sites removed. SNP filters for HWE (1e-6) and minor allele frequency (1%) were re-applied. SNP ids were updated from chr:bp to rsIDs using dbsnp_138.b37.vcf. A total of 7545595 imputed SNPs for consideration in AD-PRS analysis.

### Creation of polygenic scores

Polygenic score calculations were performed according to the procedure described by the International Schizophrenia Consortium^39^. Training data were from the most recent AD GWAS^1^. These data are publicly available from http://www.pasteur-lille.fr/en/recherche/u744/igap/igap_download.php. SNPs were removed from all analyses if they had a low minor allele frequency (P < 0.01). Subsequently, the data were pruned for linkage disequilibrium using the clumping function (–clump) in plink 1.9^35^ removing SNPs within 500 kilobase (–clump-kb) and *R*^2^ > 0.25 (–clump-r2) with a more significantly associated SNP. We used the –score command in PLINK to calculate polygenic score. For the creation of the AD-PRS, we considered SNPs that were associated with AD that surpassed the GWAS threshold (P_T_ < 5 × 10^−8^). We chose a conservative P-threshold as prior fMRI neuroimaging studies have demonstrated that conservative P-thresholds have been successful in predicting blood oxygenation level dependent (BOLD) MRI measures linked to AD^28^. As we aimed to understand the contribution of SNPs outside the *APOE* locus, we employed a conservative approach, where we removed all SNPs from chromosome 19 before creating an AD-PRS. We aimed to remove any confounding signal from variants in linkage disequilibrium (LD) with the APOE locus. A total of 17 SNPs were considered in the AD-PRS calculation. Removing the entire *APOE* locus (removing chr19: 45.053–45.73 Mb, rather than the whole of chromosome 19; N_SNPS_ = 19) did not significantly affect our results. We further created 3 additional AD-PRS at progressively liberal P-thresholds to explore optimal model performance (P_T_ < 5 × 10^−6^; P_T_ < 5 × 10^−4^; P_T_ < 5 × 10^−2^). Although our sample predominately included individuals of western European descent, we also aimed to ensure that the population stratification was not accounting for variation linked to AD-PRS. We therefore extracted the first five principle components from the linkage-disequilibrium (LD) pruned genotypes and included these as covariates in the analysis.

### Imaging procedure

Imaging data were collected on a 3 Tesla General Electric (GE) scanner. Anatomical T1-weighted images were acquired with a 3D fast spoiled gradient echo sequence (FSPGR) (parameters: 172 contiguous sagittal slices with a slice thickness of 1 mm, TR = 7.9, TE = 3 ms, inversion time of 450 ms, flip angle = 20°, a FOV of 256 × 256 × 176 mm, matrix size 256 × 256 × 192 to yield 1 mm isotropic voxel resolution images).

Resting CBF data was collected using a pseudo-continuous arterial spin labelling (PCASL) sequence^40^. The study consisted of a single MRI session (which also comprised other functional and structural scans), and the PCASL sequence lasted approximately 6 minutes. Resting-state CBF maps (rCBF) and whole brain CBF (wbCBF) measurements were created with a PCASL sequence identical to the one previously described. Parameters included a 1.5 second tag and a post-labelling delay of 1.5 seconds. The images for the PCASL sequence were acquired using a 3D fast spin echo (FSE) spiral multi-slice readout (parameters: number of excitations = 3, time to echo = 32 ms, echo time train length = 64, TR = 5.5 seconds, matrix size = 48 × 64 × 60, FOV = 18 × 23 × 18 cm).

### Grey matter CBF (gmCBF) analysis

Recent evidence suggests vascular dysfunction in AD is uniformly reduced across the brain^15^, therefore we looked at whether this is also the case in young individuals with increased cumulative risk for developing AD. This was conducted by extracting mean CBF from each grey matter image.

**Figure 3:**
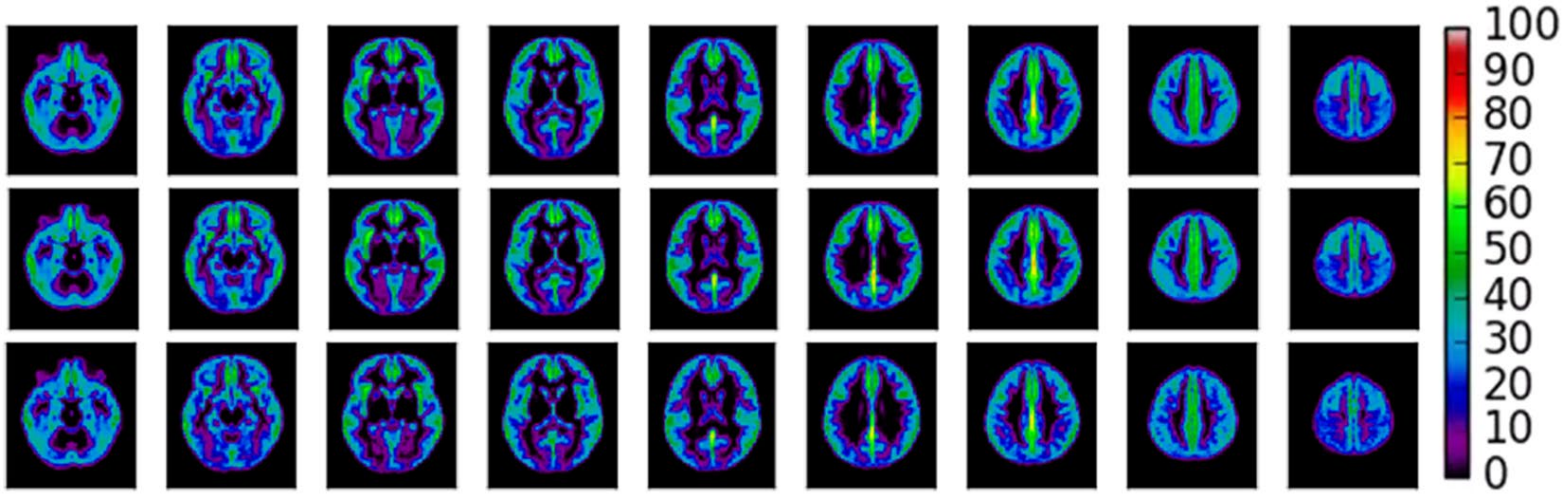
Average gmCBF maps. Top. A mean gmCBF map of all participants within the sample (N = 75). Middle. Mean gmCBF for APOE ɛ4− group (N = 54). Below. Mean gmCBF for APOE ɛ4+ group (N = 21). Colorbar represents regional gmCBF (ml/min/100 g).

Briefly, grey matter CBF was measured in native space for each of the 75 individuals. First, anatomical T1 weighted FSPGR images were registered to the M0 image acquired as part of the calibration of the CBF image acquisition, generating a transformation matrix. This transformation matrix was then applied to the skull stripped T1w anatomical (with reference/warping to the M0) using FSL’s Brain Extraction Tool^41^. From here, we used linear registration FSL’s FLIRT^42,43^ to register the skull stripped anatomical image to the M0 transformation matrix (Montreal Neurological Institute (MNI) space) and the difference was calculated between this and the subjects native space, providing data in the same space as the CBF data. The two transformation matrices for each participant were then concatenated to produce a matrix for the low resolution CBF image. All CBF images were then warped to standard MNI template using FSL’s FLIRT^41,42^. The priors for the grey matter were then registered to the skull stripped M0 image, creating a mask of each individual’s grey matter where CBF values could be extracted. Grey matter volume (GMV) was approximated as the mean intensity of the segmented grey matter image. See Fig. 3 for a) the mean gmCBF of the cohort and b) for *APOE* ɛ4- and c) *APOE* ɛ4+ .

### Voxel based morphometry

Regional grey matter density was estimated for each individual. Briefly, structural data was analysed with FSL-VBM^44^, (http://fsl.fmrib.ox.ac.uk/fsl/fslwiki/FSLVBM), an optimised VBM protocol^45^ carried out with FSL tools^42^. First, structural images were brain-extracted and grey matter-segmented before being registered to the MNI 152 standard space using non-linear registration. The resulting images were averaged and flipped along the x-axis to create a left-right symmetric, study-specific grey matter template. Second, all native grey matter images were non-linearly registered to this study-specific template and “modulated” to correct for local expansion (or contraction) due to the non-linear component of the spatial transformation. The modulated grey matter images were then smoothed with an isotropic Gaussian kernel with a sigma of 3 mm. These images were entered into same regression models as the standardised gmCBF images.

### Power analysis

Current estimates suggest that the combination of *APOE* locus and GWAS loci (N_SNPS_ = 20) classify AD cases and controls with an accuracy of 66%, assuming a lifetime prevalence of 2%^2^, equating to a moderate effect size (Cohen’s D = 0.583; r = 0.28)^46^. Based upon this anticipated effect size, we had approximately 69% power to detect an combined effect of these AD risk loci (N = 75, α = 0.05), calculated with ‘pwr’ in R^47^.

### Cognitive instruments

We assessed cognition via the MATRICS Consensus Cognitive Battery (MCCB), which measures composite cognition across seven sub-domains including working memory, attention, speed of processing, verbal and visual learning and social cognition^48^.

### Statistical analysis

Linear regression models were employed in R (https://www.r-project.org/; version 3.1.3) to assess the impact of both *APOE* and AD-PRS risk on whole brain grey matter CBF. Whole brain grey matter CBF was introduced as our dependent variable where *APOE* and AD-PRS were added together as regressors. Age, gender and grey matter volume (GMV) were included as covariates in the analyses. A post-hoc analysis was repeated using the aforementioned linear regression approach (using the same covariates) and was conducted at a voxel-wise level to explore regional effects of *APOE* and AD-PRS on gmCBF. For whole brain analysis (gmCBF and GMV), the family-wise error rate was controlled with nonparametric permutation testing (5000 permutations) and TFCE (threshold free cluster enhancement) which effectively controls for multiple comparisons, compared to cluster extent thresholding^49^.

### Approval and accordance

The study was approved by the ethics committee of the School of Psychology, Cardiff University. Methods were carried out in accordance with guidelines and regulations.

### Informed consent

Informed consent was obtained for all procedures used for this study.

## Data Availability

Analysis code and data can be made available upon request to Cardiff University Brain Research Imaging Centre.

## References

[1] Lambert JC (2013). Meta-analysis of 74,046 individuals identifies 11 new susceptibility loci for Alzheimer’s disease. Nat Genet.

[2] Escott-Price V, Shoai M, Pither R, Williams J, Hardy J (2017). Polygenic score prediction captures nearly all common genetic risk for Alzheimer’s disease. Neurobiol Aging.

[3] Escott-Price V (2015). Common polygenic variation enhances risk prediction for Alzheimer’s disease. Brain.

[4] Zhang X (2015). Bridging Integrator 1 (BIN1) Genotype Effects on Working Memory, Hippocampal Volume, and Functional Connectivity in Young Healthy Individuals. Neuropsychopharmacology.

[5] Zhang P (2015). Impacts of PICALM and CLU variants associated with Alzheimer’s disease on the functional connectivity of the hippocampus in healthy young adults. Brain structure & function.

[6] Trachtenberg AJ (2012). The effects of APOE on the functional architecture of the resting brain. Neuroimage.

[7] Filippini N (2009). Distinct patterns of brain activity in young carriers of the APOE-epsilon4 allele. Proc Natl Acad Sci USA.

[8] Lancaster TM (2015). Alzheimer’s disease risk variant in CLU is associated with neural inefficiency in healthy individuals. Alzheimer’s & dementia: the journal of the Alzheimer’s Association.

[9] Lancaster TM (2011). Neural hyperactivation in carriers of the Alzheimer’s risk variant on the clusterin gene. European neuropsychopharmacology: the journal of the European College of Neuropsychopharmacology.

[10] Foley SF (2017). Multimodal Brain Imaging Reveals Structural Differences in Alzheimer’s Disease Polygenic Risk Carriers: A Study in Healthy Young Adults. Biol Psychiatry.

[11] Prvulovic D, Van de Ven V, Sack AT, Maurer K, Linden DE (2005). Functional activation imaging in aging and dementia. Psychiatry Res.

[12] Methia N (2001). ApoE deficiency compromises the blood brain barrier especially after injury. Molecular medicine.

[13] Jones L (2010). Genetic evidence implicates the immune system and cholesterol metabolism in the aetiology of Alzheimer’s disease. PLoS One.

[14] Yip AG (2005). APOE, vascular pathology, and the AD brain. Neurology.

[15] Iturria-Medina Y (2016). Early role of vascular dysregulation on late-onset Alzheimer’s disease based on multifactorial data-driven analysis. Nature communications.

[16] Fleisher AS (2009). Cerebral perfusion and oxygenation differences in Alzheimer’s disease risk. Neurobiol Aging.

[17] Wierenga CE (2013). Interaction of age and APOE genotype on cerebral blood flow at rest. J Alzheimers Dis.

[18] Wierenga CE, Bondi MW (2007). Use of functional magnetic resonance imaging in the early identification of Alzheimer’s disease. Neuropsychology review.

[19] Suri S (2015). Reduced cerebrovascular reactivity in young adults carrying the APOE epsilon4 allele. Alzheimer’s & dementia: the journal of the Alzheimer’s Association.

[20] Wolters FJ (2017). Cerebral Perfusion and the Risk of Dementia: A Population-Based Study. Circulation.

[21] Filippini N (2011). Differential effects of the APOE genotype on brain function across the lifespan. Neuroimage.

[22] Kim SM (2013). Regional cerebral perfusion in patients with Alzheimer’s disease and mild cognitive impairment: effect of APOE epsilon4 allele. Neuroradiology.

[23] Chao LL (2010). ASL perfusion MRI predicts cognitive decline and conversion from MCI to dementia. Alzheimer disease and associated disorders.

[24] Biffi A (2010). Genetic variation and neuroimaging measures in Alzheimer disease. Arch Neurol.

[25] Harrison, T. M. *et al*. An Alzheimer’s Disease Genetic Risk Score Predicts Longitudinal Thinning of Hippocampal Complex Subregions in Healthy Older Adults. *eNeuro* **3**, 10.1523/ENEURO.0098-16.2016 (2016). 10.1523/ENEURO.0098-16.2016 PMC4945997 27482534

[26] Mormino EC (2016). Polygenic risk of Alzheimer disease is associated with early- and late-life processes. Neurology.

[27] Lupton MK (2016). The effect of increased genetic risk for Alzheimer’s disease on hippocampal and amygdala volume. Neurobiol Aging.

[28] Xiao E (2017). Late-Onset Alzheimer’s Disease Polygenic Risk Profile Score Predicts Hippocampal Function. Biological psychiatry. Cognitive neuroscience and neuroimaging.

[29] Germuska M, Bulte DP (2014). MRI measurement of oxygen extraction fraction, mean vessel size and cerebral blood volume using serial hyperoxia and hypercapnia. Neuroimage.

[30] Jain V, Langham MC, Wehrli FW (2010). MRI estimation of global brain oxygen consumption rate. Journal of cerebral blood flow and metabolism: official journal of the International Society of Cerebral Blood Flow and Metabolism.

[31] Wise RG, Harris AD, Stone AJ, Murphy K (2013). Measurement of OEF and absolute CMRO2: MRI-based methods using interleaved and combined hypercapnia and hyperoxia. Neuroimage.

[32] Germuska M (2018). Dual-calibrated fMRI measurement of absolute cerebral metabolic rate of oxygen consumption and effective oxygen diffusivity. Neuroimage.

[33] Lucas SJ, Cotter JD, Brassard P, Bailey DM (2015). High-intensity interval exercise and cerebrovascular health: curiosity, cause, and consequence. Journal of cerebral blood flow and metabolism: official journal of the International Society of Cerebral Blood Flow and Metabolism.

[34] Wightman EL (2015). Dietary nitrate modulates cerebral blood flow parameters and cognitive performance in humans: A double-blind, placebo-controlled, crossover investigation. Physiology & behavior.

[35] Chang CC (2015). Second-generation PLINK: rising to the challenge of larger and richer datasets. GigaScience.

[36] McCarthy S (2016). A reference panel of 64,976 haplotypes for genotype imputation. Nat Genet.

[37] Loh PR (2016). Reference-based phasing using the Haplotype Reference Consortium panel. Nat Genet.

[38] Das S (2016). Next-generation genotype imputation service and methods. Nat Genet.

[39] International Schizophrenia C (2009). Common polygenic variation contributes to risk of schizophrenia and bipolar disorder. Nature.

[40] Dai W, Garcia D, de Bazelaire C, Alsop DC (2008). Continuous flow-driven inversion for arterial spin labeling using pulsed radio frequency and gradient fields. Magnetic resonance in medicine: official journal of the Society of Magnetic Resonance in Medicine/Society of Magnetic Resonance in Medicine.

[41] Smith SM (2002). Fast robust automated brain extraction. Hum Brain Mapp.

[42] Smith SM (2004). Advances in functional and structural MR image analysis and implementation as FSL. Neuroimage.

[43] Jenkinson M, Bannister P, Brady M, Smith S (2002). Improved optimization for the robust and accurate linear registration and motion correction of brain images. Neuroimage.

[44] Douaud G (2007). Anatomically related grey and white matter abnormalities in adolescent-onset schizophrenia. Brain.

[45] Good CD (2001). A voxel-based morphometric study of ageing in 465 normal adult human brains. Neuroimage.

[46] Ruscio J (2008). A probability-based measure of effect size: robustness to base rates and other factors. Psychological methods.

[47] Champely, S. Pwr: Basic functions for power analysis. R package version 1.1.1, http://CRAN.R-project.org/package=pwr (2012).

[48] Nuechterlein KH (2008). The MATRICS Consensus Cognitive Battery, part 1: test selection, reliability, and validity. Am J Psychiatry.

[49] Smith SM, Nichols TE (2009). Threshold-free cluster enhancement: addressing problems of smoothing, threshold dependence and localisation in cluster inference. Neuroimage.

